# Rhabdomyolysis among hospitalized patients for salicylate intoxication in the United States: Nationwide inpatient sample 2003–2014

**DOI:** 10.1371/journal.pone.0248242

**Published:** 2021-03-08

**Authors:** Wisit Kaewput, Charat Thongprayoon, Tananchai Petnak, Wisit Cheungpasitporn, Fawad Qureshi, Boonphiphop Boonpheng, Saraschandra Vallabhajosyula, Tarun Bathini, Sohail Abdul Salim, Tibor Fülöp

**Affiliations:** 1 Department of Military and Community Medicine, Phramongkutklao College of Medicine, Bangkok, Thailand; 2 Division of Nephrology and Hypertension, Mayo Clinic, Rochester, Minnesota, United States of America; 3 Division of Pulmonary and Critical Care Medicine, Faculty of Medicine, Ramathibodi Hospital, Mahidol University, Bangkok, Thailand; 4 Division of Pulmonary and Critical Care Medicine, Department of Medicine, Mayo Clinic, Rochester, Minnesota, United States of America; 5 Department of Medicine, David Geffen School of Medicine, University of California, Los Angeles, Los Angeles, California, United States of America; 6 Section of Interventional Cardiology, Division of Cardiovascular Medicine, Department of Medicine, Emory University School of Medicine, Atlanta, Georgia, United States of America; 7 Department of Internal Medicine, University of Arizona, Tucson, Arizona, United States of America; 8 Division of Nephrology, Department of Medicine, University of Mississippi Medical Center, Jackson, Mississippi, United States of America; 9 Department of Internal Medicine, Division of Nephrology, Medical University of South Carolina, Charleston, South Carolina, United States of America; 10 Ralph H. Johnson Veterans Affairs Medical Center, Charleston, South Carolina, United States of America; Ospedale Sant’Antonio, ITALY

## Abstract

**Introduction:**

This study aimed to assess the risk factors and impact of rhabdomyolysis on treatments, outcomes, and resource utilization in hospitalized patients for salicylate intoxication in the United States.

**Materials and methods:**

The National Inpatient Sample was utilized to identify hospitalized patients with a primary diagnosis of salicylate intoxication from 2003–2014. Rhabdomyolysis was identified using hospital diagnosis code. We compared the clinical characteristics, in-hospital treatment, outcomes, and resource utilization between patients with and without rhabdomyolysis.

**Results:**

A total of 13,805 hospital admissions for salicylate intoxication were studied. Of these, rhabdomyolysis developed in 258 (1.9%) admissions. The risk factors for rhabdomyolysis were age>20 years, male sex, volume depletion, hypokalemia, sepsis, and seizure. After adjustment for baseline clinical characteristics, salicylate intoxication patients with rhabdomyolysis required more invasive mechanical ventilation, and renal replacement therapy. Rhabdomyolysis was significantly associated with higher risk of failure of any organ systems, and in-hospital mortality. Length of hospital stay and hospitalization cost were higher when rhabdomyolysis occurred during hospital stay.

**Conclusions:**

Rhabdomyolysis was not common in hospitalized patients for salicylate intoxication but it was associated with increased morbidity, mortality, and resource utilization.

## Introduction

Salicylate has been well-known as an analgesic and antipyretic medication for several years. Nowadays, salicylates (e.g., aspirin) are widely used as antiplatelet agents. Another form of salicylate is methyl salicylate, which can be found in tropical products, such as ointment, lotion, and herbal oil [1]. Regarding drug availability, most salicylate products are over the counter and ready to access. Hence, salicylate has become one of the most common causes of drug intoxications in the United States. In 2018, the burden of salicylate intoxication was demonstrated by the American Association of Poison Control Center: more than 25000 patients in the United States suffered from salicylate intoxication, including acetylsalicylate and methyl salicylate, with a mortality rate of 0.4%. Among these exposures, half of them were intentional ingestion [2].

When salicylate dose becomes toxic, direct stimulation to the respiratory center is exerted, leading to increased minute ventilation and respiratory alkalosis [1]. Uncoupling of oxidative mitochondrial respiration is the principal pathogenesis of salicylate intoxication. The low intracellular adenosine triphosphate (ATP) concentration causes anaerobic ATP production through glycolysis and ketogenesis, resulting in lactic and ketogenic acidosis, which mainly contributes to the wide anion gap metabolic acidosis in salicylate intoxication. The compensatory body catabolism leads to an increase in heat production, causing fever and sweating. Transient hyperglycemia may develop due to increase in glycolysis along with the decrease of glucose use. However, hypoglycemia becomes predominant in the latter stage since glycogen storage is depleted [3].

The neuromuscular effect of salicylate is uncommon. Rhabdomyolysis has been infrequently reported as a complication of salicylate intoxication [4, 5]. The specific mechanism of rhabdomyolysis in salicylate intoxication is still unclear. The uncoupling of oxidative phosphorylation leading to increase in heat production might be responsible the development of rhabdomyolysis [4]. Also, severe systemic inflammation and muscle rigidity might occur in salicylate intoxication, resulting in further heat production and muscle injury [3, 5]. Since the cells break down, intracellular electrolytes, e.g., potassium, and phosphate, release into the circulation causing hyperkalemia and hyperphosphatemia. In addition to electrolyte abnormalities, other complications of rhabdomyolysis include cardiac arrhythmia, compartment syndrome, acute kidney injury, and disseminated intravascular coagulation. The principle of rhabdomyolysis treatment includes fluid resuscitation and correct the reversible causes of rhabdomyolysis. Urinary alkalization is also considered to reduce the nephrotoxicity from myoglobinuria [6].

Although rhabdomyolysis in salicylate intoxication has not been reported frequently, its impact on clinical outcomes is considerable [4, 5]. However, the evidence from the literature is still limited on this topic [7, 8]. We conducted a large retrospective cohort study aiming to assess the risk factors and impact of rhabdomyolysis on treatments, outcomes, and resource utilization in hospitalized patients for salicylate intoxication in the United States.

## Materials and methods

### Data source

This cohort study utilized the National Inpatient Sample (NIS) database, which is the largest all-payer inpatient database in the United States. This NIS database is managed by the Healthcare Cost and Utilization Project (HCUP) under the sponsorship of the Agency for Healthcare Research and Quality (AHRQ). The project builds on the data collection efforts of State data organizations, hospital associations, and private data organizations (known as "HCUP Partners"). AHRQ transforms administrative health care data acquired from HCUP Partners into research-ready, uniform databases with a common set of data elements. The NIS database contains hospitalization data from a 20% stratified sample of hospitals in the United States. The patient-level information includes diagnosis and procedure codes. A stratified sampling approach was applied to select a nationally representational sample of hospitals in the United States. The sample of hospitals were stratified into five strata according to (1) Geographic Region—Northeast, Midwest, West, and South; (2) Control—public, private not-for-profit, and proprietary; (3) Location—urban or rural; (4) Teaching Status—teaching or non-teaching, (5) Bed Size-small, medium, and large. The Mayo Clinic institutional review board approval was waived (45 CFR 46.104d, category 4) as the data was from a de-identified public database (IRB Application #: 20–012676).

### Study population and outcomes

Patients who were admitted to hospitals from 2003 to 2014 with a primary diagnosis of salicylate intoxication, based on the International Classification of Diseases, Ninth Revision, Clinical Modification (ICD-9 CM) diagnosis code 965.1, were included. Salicylate intoxication patients were grouped based on the development of rhabdomyolysis during the hospital course.

The outcome of interest was rhabdomyolysis identified by the ICD-9 diagnosis 728.88. Our primary outcome was incidence of rhabdomyolysis in hospitalized salicylate intoxication. Secondary outcomes were to identify risk factors for rhabdomyolysis and impact of rhabdomyolysis on in-hospital treatments and outcomes.

### Data collection

Patient clinical characteristics that were collected for the study consisted of age, sex, race, year of hospitalization, alcohol use, analgesics overdose, psychotropic medication overdose, and certain comorbidities (obesity, anemia, diabetes mellitus, hypertension, dyslipidemia, coronary artery disease, congestive heart failure, atrial fibrillation/flutter, and chronic kidney disease) and acute conditions (volume depletion, hypokalemia, sepsis, seizure). Treatments that were reported in the study consisted of invasive mechanical ventilation, blood component transfusion, and renal replacement therapy. Outcomes consisted of organ failure (renal failure, respiratory failure, circulatory failure, liver failure, neurological failure, hematological failure) [9], and in-hospital mortality. Resource utilization consisted of length of hospital stay and hospitalization cost. Clinical characteristics, treatments, and outcomes during hospitalization were identified using ICD-9 codes **(S1 Table)**.

### Statistical analysis

The Shapiro-Wilk test was used for evaluating whether the observations deviated from the normal distribution. Clinical characteristics, treatments, outcomes, and resource utilization between salicylate intoxication patients with and without rhabdomyolysis were compared using student’s t-test for continuous variables, and Chi-squared test for categorical variables. Multivariable logistic regression with backward stepwise selection was performed to identify clinical characteristics independently associated with the development of rhabdomyolysis **(Table 2)**. The selection of associated factors and confounders to be included in multivariable models was based on a p<0.05 in the univariate analysis. Removal testing is based on the probability of the Wald statistic **(S2 Table)**. The association of rhabdomyolysis with clinical outcomes was assessed using logistic regression analysis, and the association with resource utilization was assessed using linear regression analysis. The analysis was adjusted for pre-specified clinical characteristics **(Table 3)**. A procedure for variable selection in which all possible confounders are entered in a single step (Enter). The pre-specified clinical characteristics in the statistic models composed of age, sex, race, the NIS year, alcohol drinking, anemia, hypertension, dyslipidemia, coronary artery disease, congestive heart failure, atrial flutter/ fibrillation, chronic kidney disease, volume depletion, hypokalemia, sepsis, and seizure. The model fit and multicollinearity test for logistic regression were assessed by -2 Log likelihood, Cox and Snell R square, Nagelkerke R square, Hosmer and Lemeshow Chi-square **(S2–S4 Tables)**. The multicollinearity for linear regression was assessed by Tolerance and Variance Inflation Factor **(S5 Table)**. Analysis was statistically significant when two-tailed p-value <0.05. SPSS statistical software (version 22.0, IBM Corporation, Armonk, NY, USA) was used for all analyses.

## Results

### Incidence of and risk factors for rhabdomyolysis

**Fig 1:**
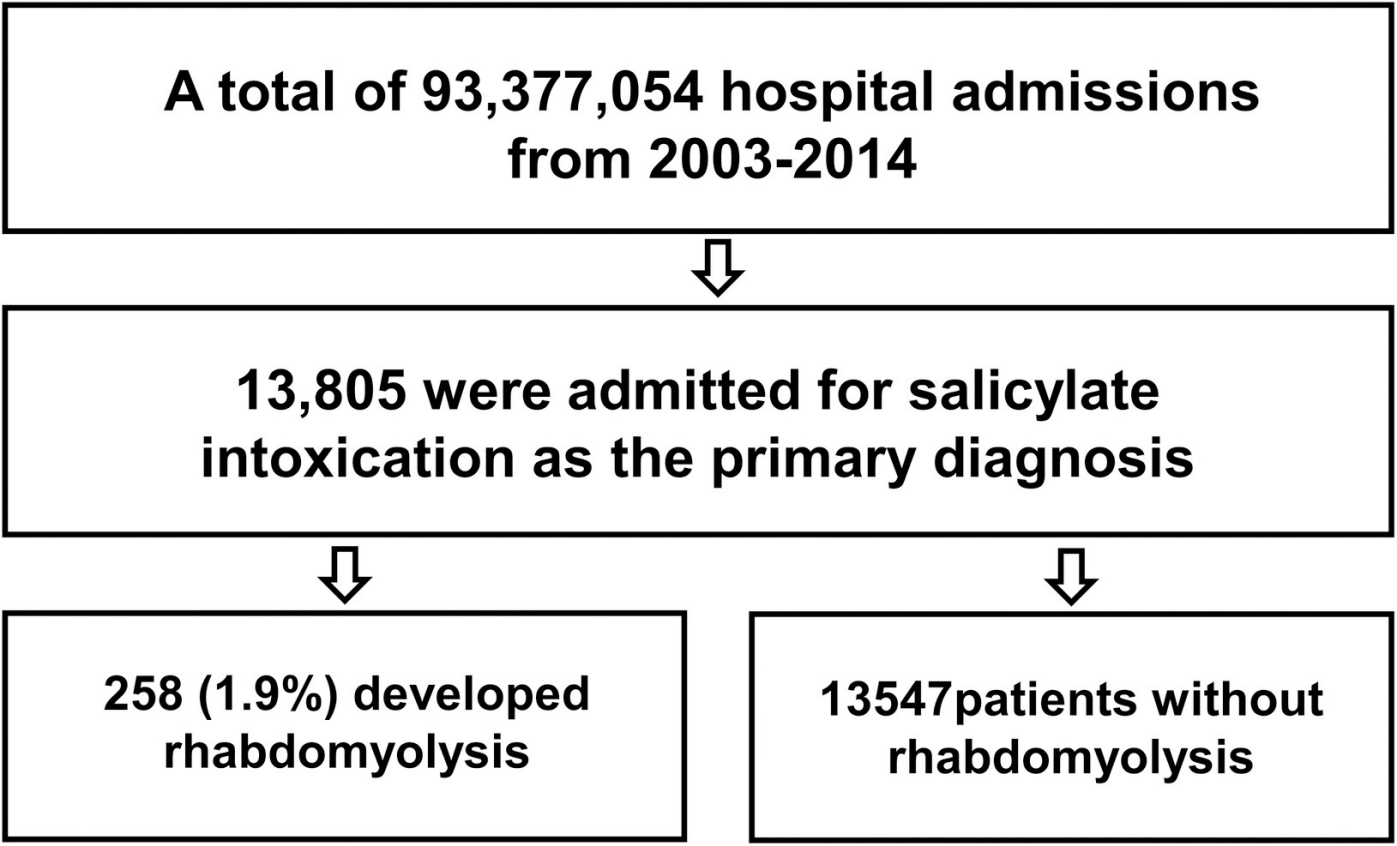
Flowchart of participants.

A total of 13,805 patients were hospitalized with a primary diagnosis of salicylate intoxication. Of these, 258 (1.9%) developed rhabdomyolysis **(Fig 1)**. **Table 1** compared clinical characteristics, treatments, outcomes, and resource utilization between patients with and without rhabdomyolysis. Multivariable analysis identified age greater than 20 years old (OR 2.36 for age 20–29 years; p = 0.02, 5.18 for age 30–39 years; p<0.001, and 10.23 for age greater than or equal to 40 years old; p<0.001), male sex (OR 1.99; p<0.001), volume depletion (OR 2.25; p<0.001), hypokalemia (OR 1.67; p<0.001), sepsis (OR 4.45; p<0.001), and seizure (OR 1.78; p = 0.01) as risk factors for the development of rhabdomyolysis **(Table 2)**.

**Table 1:** Clinical characteristics, treatments, outcomes, and resource utilization in salicylate intoxication patients with and without rhabdomyolysis.

	Total	Rhabdomyolysis	No rhabdomyolysis	P-value
Clinical characteristics				
N (%)	13805	258	13547	
Age (years)	34.0±18.7	49.2±16.9	33.7±18.6	<0.001
<20	3902 (28.3)	11 (4.3)	3891 (28.8)	<0.001
20–29	3228 (23.4)	26 (10.1)	3202 (23.7)	
30–39	1951 (14.1)	36 (14.0)	1915 (14.2)	
≥40	4710 (34.2)	185 (71.7)	4525 (33.4)	
Male	4811 (35.0)	136 (52.7)	4675 (34.6)	<0.001
Race				0.05
Caucasian	7729 (56.0)	160 (62.0)	7569 (55.9)	
African American	1391 (10.1)	31 (12.0)	1360 (10.0)	
Hispanic	1311 (9.5)	20 (7.8)	1291 (9.5)	
Other	3374 (24.4)	47 (18.2)	3327 (24.6)	
The NIS year				
2003–2008	7341 (53.2)	114 (44.2)	7227 (53.3)	0.003
2009–2014	6464 (46.8)	144 (55.8)	6320 (46.7)	
Alcohol drinking	2216 (16.1)	60 (23.3)	2156 (15.9)	0.001
Concurrent analgesics overdose	967 (7.0)	20 (7.8)	947 (7.0)	0.64
Concurrent psychotropic medication overdose	566 (4.1)	12 (4.7)	554 (4.1)	0.65
Obesity	521 (3.8)	12 (4.7)	509 (3.8)	0.46
Anemia	897 (6.5)	30 (11.6)	867 (6.4)	0.001
Diabetes mellitus	801 (5.8)	21 (8.1)	780 (5.8)	0.11
Hypertension	2137 (15.5)	76 (29.5)	2061 (15.2)	<0.001
Dyslipidemia	749 (5.4)	28 (10.9)	721 (5.3)	<0.001
Coronary artery disease	512 (3.7)	21 (8.1)	491 (3.6)	<0.001
Congestive heart failure	239 (1.7)	17 (6.6)	222 (1.6)	<0.001
Atrial flutter/fibrillation	172 (1.2)	14 (5.4)	158 (1.2)	<0.001
Chronic kidney disease	218 (1.6)	*	208 (1.5)	0.008
Volume depletion	739 (5.4)	39 (15.1)	700 (5.2)	<0.001
Hypokalemia	3508 (25.4)	96 (37.2)	3412 (25.2)	<0.001
Sepsis	126 (0.9)	17 (6.6)	109 (0.8)	<0.001
Seizure	565 (4.1)	24 (9.3)	541 (4.0)	<0.001
Treatments				
Invasive mechanical ventilation	760 (5.5)	88 (34.1)	672 (5.0)	<0.001
Blood component transfusion	356 (2.6)	17 (6.6)	339 (2.5)	<0.001
Renal replacement therapy	811 (5.9)	71 (27.5)	740 (5.5)	<0.001
Complications and outcomes				
Renal failure	1279 (9.3)	127 (49.2)	1152 (8.5)	<0.001
Respiratory failure	943 (6.8)	100 (38.8)	843 (6.2)	<0.001
Circulatory failure	484 (3.5)	33 (12.8)	451 (3.3)	<0.001
Liver failure	110 (0.8)	19 (7.4)	91 (0.7)	<0.001
Neurological failure	689 (5.0)	42 (16.3)	647 (4.8)	<0.001
Hematological failure	303 (2.2)	30 (11.6)	273 (2.0)	<0.001
In-hospital mortality	132 (1.0)	13 (5.0)	119 (0.9)	<0.001
Resource utilization				
Length of hospital stay (days)	2.6±3.3	7.3±7.8	2.5±3.1	<0.001
Hospitalization cost ($)	18128±29613	58321±79137	17354± 27241	<0.001

Continuous variables are reported as mean ± standard deviation; categorical as counts (percentages), *the number is below the cut-off allowed to be reported per HCUP/NIS regulations.

**Table 2:** Univariable and multivariable analysis assessing factors associated with rhabdomyolysis in salicylate intoxication patients.

Variables	Univariable analysis		Multivariable analysis^a^	
	Crude odds ratio (95%CI)	P-value	Adjusted odds ratio (95%CI)	P-value
Age (years)				
<20	1 (reference)		1 (reference)	
20–29	2.87 (1.42–5.82)	0.003	2.36 (1.16–4.81)	0.02
30–39	6.65 (3.38–13.09)	<0.001	5.18 (2.62–10.24)	<0.001
≥40	14.46 (7.86–26.61)	<0.001	10.23 (5.53–18.95)	<0.001
Male	2.11 (1.65–2.70)	<0.001	1.99 (1.54–2.56)	<0.001
Race				
Caucasian	1 (reference)			
African American	1.08 (0.73–1.59)	0.70		
Hispanic	0.73 (0.46–1.17)	0.19		
Other	0.67 (0.48–0.93)	0.02		
The NIS year				
2003–2008	1 (reference)			
2009–2014	1.44 (1.13–1.85)	0.004		
Alcohol drinking	1.60 (1.20–2.15)	0.002		
Concurrent analgesics overdose	1.12 (0.71–1.77)	0.64		
Concurrent psychotropic medication overdose	1.14 (0.64–2.06)	0.65		
Obesity	1.25 (0.70–2.25)	0.46		
Anemia	1.92 (1.31–2.83)	0.001		
Diabetes Mellitus	1.45 (0.92–2.28)	0.11		
Hypertension	2.33 (1.77–3.05)	<0.001		
Dyslipidemia	2.17 (1.45–3.23)	<0.001		
Coronary artery disease	2.36 (1.49–3.72)	<0.001		
Congestive heart failure	4.23 (2.54–7.05)	<0.001	1.69 (0.98–2.91)	0.06
Atrial flutter/fibrillation	4.86 (2.78–8.52)	<0.001	1.77 (0.98–3.21)	0.06
Chronic kidney disease	2.59 (1.35–4.94)	0.004		
Volume depletion	3.27 (2.31–4.63)	<0.001	2.25 (1.57–3.22)	<0.001
Hypokalemia	1.76 (1.36–2.27)	<0.001	1.67 (1.28–2.17)	<0.001
Sepsis	8.70 (5.14–14.73)	<0.001	4.45 (2.57–7.72)	<0.001
Seizure	2.47 (1.61–3.79)	<0.001	1.78 (1.15–2.76)	0.01

^a^Adjusted analysis using multiple logistic regression with backward stepwise (Wald) selection method.

### Impact of rhabdomyolysis on in-hospital treatments, and outcomes

According to the adjusted analyses, salicylate intoxication patients with rhabdomyolysis were significantly more exposed to invasive mechanical ventilation (OR 5.76; p<0.001), and renal replacement therapy (OR 3.65; p<0.001). In addition, rhabdomyolysis was significantly associated with increased risk of renal failure (6.04; p<0.001), respiratory failure (OR 5.40; p<0.001), circulatory failure (OR 2.05; p = 0.001), liver failure (OR 6.19; p<0.001), neurological failure (OR 2.41; p<0.001), hematological failure (OR 3.19; p<0.001), and in-hospital mortality (OR 2.92; p = 0.001) **(Table 3)**.

**Table 3:** The association between rhabdomyolysis and in-hospital treatment, outcomes, and resource utilization in salicylate intoxication patients.

Variables	Univariable analysis		Multivariable analysis^a^	
	Crude odds ratio (95%CI)	P-value	Adjusted odds ratio (95%CI)	P-value
Treatments				
Invasive mechanical ventilation	9.92 (7.58–12.98)	<0.001	5.76 (4.27–7.77)	<0.001
Blood component transfusion	2.75 (1.66–4.55)	<0.001	1.20 (0.69–2.08)	0.52
Renal replacement therapy	6.57 (4.95–8.72)	<0.001	3.65 (2.70–4.94)	<0.001
Complications and outcomes				
Renal failure	10.43 (8.11–13.41)	<0.001	6.04 (4.55–8.01)	<0.001
Respiratory failure	9.54 (7.35–12.37)	<0.001	5.40 (4.05–7.20)	<0.001
Circulatory failure	4.26 (2.92–6.21)	<0.001	2.05 (1.36–3.11)	0.001
Liver failure	11.76 (7.05–19.59)	<0.001	6.19 (3.59–10.68)	<0.001
Neurological failure	3.88 (2.76–5.45)	<0.001	2.41 (1.70–3.43)	<0.001
Hematological failure	6.40 (4.29–9.54)	<0.001	3.19 (2.09–4.87)	<0.001
In-hospital mortality	5.98 (3.32–10.74)	<0.001	2.92 (1.55–5.49)	0.001
Resource utilizations	Coefficient (95% CI)	P-value	Adjusted co efficient (95% CI)	P-value
Length of hospital stay (days)	4.9 (4.5–5.2)	<0.001	3.4 (3.1–3.8)	<0.001
Hospitalization cost ($)	40966.5 (37376.3–44556.7)	<0.001	28822.1 (25533.3–32111.0)	<0.001

^a^Adjusted for age, sex, race, the national inpatient sample (NIS) year, alcohol drinking, anemia, hypertension, dyslipidemia, coronary artery disease, congestive heart failure, atrial flutter/fibrillation, chronic kidney disease, volume depletion, hypokalemia, sepsis, and seizure.

### Impact of rhabdomyolysis on resource utilization

In salicylate intoxication patients with rhabdomyolysis, the mean length of hospital stay increased by 3.4 days (p<0.001), and the mean hospitalization cost increased by $28822.1 (p<0.001) compared with patients without rhabdomyolysis (**Table 3**). The goodness-of-fit tests showed the model adequately fits the data **(S2–S4 Tables)**. The collinearity diagnostics for linear regression showed that there is no multicollinearity **(S5 Table)**.

## Discussion

This study is a large retrospective cohort study of salicylate intoxication focusing on risk factors for rhabdomyolysis and its impact on outcomes and resource utilization. We observed that age greater than 20 years, sepsis, volume depletion, male gender, seizure, and hypokalemia increased the risk of rhabdomyolysis among patients suffering from salicylate intoxication. Rhabdomyolysis was associated with higher in-hospital mortality and requirement of invasive mechanical ventilation and renal replacement therapy. Furthermore, rhabdomyolysis was associated with an increased risk of organ failures. Finally, salicylate intoxication patients with rhabdomyolysis had significantly longer length of hospital stay and higher hospitalization costs.

The prevalence of rhabdomyolysis in patients with salicylate intoxication requiring hospitalization in our study was 1.9%. Since rhabdomyolysis is not a common complication in salicylate intoxications, the incidence of rhabdomyolysis has not been reported. The risk of rhabdomyolysis progressively increased with increasing age. Several factors might precipitate rhabdomyolysis in elderly patients, including drug interactions due to polypharmacy and immobilization [6, 10]. Sepsis has also been associated with rhabdomyolysis [11, 12]. Most sepsis patients developing rhabdomyolysis have multiple causes of rhabdomyolysis, such as immobilization, hypokalemia, statin use [11]. The suspected rhabdomyolysis mechanisms include direct muscle infection, toxin production, cytokine-induced muscle injury, and muscle ischemia, particularly in shock state [11].

Volume depletion is common in salicylate intoxication. Decrease of intravascular volume may result in higher risk of muscle ischemia and enhance the degree of muscle injury in salicylate intoxication. Besides, our study observed an increased risk of rhabdomyolysis among male patients. Previous studies also reported the association between male gender and increased risk of rhabdomyolysis [13, 14]. Higher baseline creatine kinase in male gender might contribute to the higher proportion of male gender in rhabdomyolysis [15, 16]. Other risk factors of rhabdomyolysis demonstrated in our study included seizure and hypokalemia, which are well-known causes of rhabdomyolysis [6].

Rhabdomyolysis increased the risk of in-hospital mortality in salicylate intoxication. Several life-threatening complications, such as electrolyte abnormalities and cardiac arrhythmia, may develop in rhabdomyolysis and enhance the disease severity of salicylate intoxication leading to worse outcomes [13]. Regarding hospitalized complications, rhabdomyolysis was associated with an increased risk of end-organ failures. Risk of renal failure in rhabdomyolysis has been well described. Intravascular fluid depletion due to intramuscular fluid sequestration, renal vasoconstriction from cytokine release, and tubular obstruction from myoglobin are responsible for renal failure in rhabdomyolysis [14, 17]. In contrast to renal failure, there is no research focusing on the direct effect of myoglobin on the liver. Nevertheless, an indirect systemic effect from cytokine release after muscle injury might be responsible for the liver injury in rhabdomyolysis [18]. Besides, renal failure, a common complication of rhabdomyolysis, leads to the reduction of salicylate clearance during supratherapeutic dose may enhance the severity of salicylate intoxication and direct damage to the liver [19]. Finally, salicylates stimulate respiratory center resulting in hyperventilation. However, the respiratory reserve may not be adequate if the respiratory drive is concomitantly enhanced by other metabolic stimuli, such as metabolic acidosis due to rhabdomyolysis, leading to respiratory failure.

Our study provided patients with salicylate intoxication who were in risk for rhabdomyolysis. According to non-specific signs and symptoms of rhabdomyolysis, physicians should keep rhabdomyolysis in the differential diagnosis, particularly in high-risk patients. The early diagnosis and prompt treatment might prevent complications and improve outcome.

There are some limitations in our study. The NIS is a hospitalized database. Therefore, we did not evaluate the long-term outcomes of rhabdomyolysis after salicylate intoxication. Second, our study did not include some potential variables, such as creatine kinase levels and salicylate levels, since these data were limited in the database. These factors may impact on the severity of diseases and outcomes. Third, due to a lack of timing when condition occurred during hospital course, we could not conclude which condition was the cause or consequence of other complications. Our study could not assess temporal association between conditions. Besides, we could not use rhabdomyolysis as the early marker to predict the worse outcome. Fourth, the diagnosis of rhabdomyolysis was based on ICD codes, not on serum creatine levels. Therefore, the disease may be heterogeneous due to the lack of the diagnostic criteria. Study subjects might have various severity ranging from isolated increased creatine kinase to classical manifestation of rhabdomyolysis. Finally, the data collection and analysis were limited by the data included in the database, which may have not been thorough and comprehensive, and some potentially interesting data may have been missing.

## Conclusions

Although rhabdomyolysis is uncommon after salicylate intoxication, it was associated with increased in-hospital mortality, end-organ failures, resource utilization, and length of stay. Factors associated with increased risk of rhabdomyolysis included age greater than 20 years old, sepsis, volume depletion, male gender, seizure, and hypokalemia.

## Supporting information

S1 Checklist STROBE statement—checklist of items that should be included in reports of observational studies. (DOCX)

S1 Table ICD 9 CM codes. (DOCX)

S2 Table Step of variables selection by backward stepwise method. (DOCX)

S3 Table The tests to assess model fit for multiple logistic regression. (DOCX)

S4 Table The tests to assess model fit for multiple linear regression. (DOCX)

S5 Table The collinearity test. (DOCX)
