# Supplementary material for: Rhabdomyolysis among hospitalized patients for salicylate intoxication in the United States: Nationwide inpatient sample 2003–2014
Source: PLoS One. 2021 Mar 8;16(3):e0248242. doi: 10.1371/journal.pone.0248242 (PMC7939294; doi:10.1371/journal.pone.0248242)
Supplement: S1 Table — (DOCX) [file pone.0248242.s002.docx]

**S1 Table:** ICD 9 CM codes

| Salicylate poisoning | 965.1 |
| --- | --- |
| Alcohol drinking | 291.0, 291.1, 291.2, 291.3, 291.4, 291.5, 291.8, 291.81, 291.82, 291.89, 303.00-303.03, 303.90-303.93, 305.00-305.03 |
| Poisoning by aromatic analgesics | 965.4 |
| Psychotropic agents’ overdose | 969.xx |
| Anemia | 283.00-285.9 |
| Obesity | 278.0, 278.00, 278.01, 649.10– 649.14, 793.91, V85.30–V85.4, V85.54 |
| Diabetes Mellitus | 249.00–249.31, 250.00–250.33, 648.00–648.04, 249.40–249.91, 250.40–250.93, 775.1 |
| Hypertension | 401.1, 401.9, 642.00–642.24, 401.0, 402.00– 405.99, 437.2, 642.10–642.24, 642.70–642.94 |
| Dyslipidemia | 272.xx |
| Congestive heart failure | 428.xx |
| Chronic kidney disease | 585.1, 585.2, 585.3, 585.3, 585.4, 585.5, 585.6, 585.9 |
| Atrial flutter/fibrillation | 427.31, 427.32 |
| Coronary artery disease | 410.xx, 411.xx, 412.xx, 413.xx, 414.xx |
| Invasive mechanical ventilation | 96.70-96.73 |
| Renal replacement therapy | 39.95, v45.1, v56.0, v56.1, 54.98, v56.2, v56.32 |
| Blood transfusion | 99.00-99.07 |
| Hypokalemia | 276.8 |
| Volume depletion disorder | 276.5, 276.51, 276.52 |
| Seizure | 345.00-345.91, 780.3-780.39, 89.14 |
| Rhabdomyolysis | 728.88 |
| Sepsis | 003.1, 003.21, 020.2, 022.3, 036.0, 036.1, 036.2, 036.3, 036.42, 038.0, 038.10, 038.11, 038.19, 038.2, 038.3, 038.4, 038.40, 038.41, 038.42, 038.43, 038.44, 038.49, 038.8, 038.9, 098.82, 098.84, 098.89, 112.5, 112.81, 112.83, 115.04, 115.14, 115.94, 117.9, 785.52, 790.7, 995.92 |
| Acute kidney injury | 584, 584.5, 584.6, 584.7, 584.8, 584.9 (exclude 585.5, 585.6) |
| Respiratory failure | 518.81, 518.82, 518.85, 786.09, 799.1, 96.7, 96.70, 96.71, 96.72 |
| Circulatory failure | 458.8, 458.9, 785.5, 785.50, 785.51, 785.52, 785.59, 796.3 |
| Liver failure | 570, 572.2, 573.3, 573.4 |
| Neurological failure | 293, 293.0, 293.1, 293.8, 293.81, 293.82, 293.83, 293.84, 293.89, 293.9, 348.1, 348.3, 348.30, 348.31, 780.01, 780.09, 48.39, 89.14 |
| Hematological failure | 286.6, 286.7, 286.9, 287.49, 287.5 |
