# Supplementary material for: Rhabdomyolysis among hospitalized patients for salicylate intoxication in the United States: Nationwide inpatient sample 2003–2014
Source: PLoS One. 2021 Mar 8;16(3):e0248242. doi: 10.1371/journal.pone.0248242 (PMC7939294; doi:10.1371/journal.pone.0248242)
Supplement: S2 Table — (DOCX) [file pone.0248242.s003.docx]

**S2 Table** Step of variables selection by backward stepwise method

| Variables | Step1 | Step2 | Step3 | Step4 | Step5 | Step6 | Step7 | Step8 | Step9 |
| --- | --- | --- | --- | --- | --- | --- | --- | --- | --- |
| Age |  |  |  |  |  |  |  |  |  |
| Sex |  |  |  |  |  |  |  |  |  |
| Race |  |  |  |  |  |  |  |  | X |
| The NIS year |  |  |  |  |  |  | X | X | X |
| Alcohol drinking |  |  |  |  |  |  |  | X | X |
| Anemia |  |  |  |  | X | X | X | X | X |
| Hypertension |  |  | X | X | X | X | X | X | X |
| Dyslipidemia |  |  |  | X | X | X | X | X | X |
| Coronary artery disease |  |  |  |  |  | X | X | X | X |
| Congestive heart failure |  |  |  |  |  |  |  |  |  |
| Atrial flutter/fibrillation |  |  |  |  |  |  |  |  |  |
| Chronic kidney disease |  | X | X | X | X | X | X | X | X |
| Volume depletion |  |  |  |  |  |  |  |  |  |
| Hypokalemia |  |  |  |  |  |  |  |  |  |
| Sepsis |  |  |  |  |  |  |  |  |  |
| Seizure |  |  |  |  |  |  |  |  |  |
| Wald (Constant) | 298.38 | 298.43 | 298.38 | 298.35 | 298.43 | 298.97 | 305.34 | 304.37 | 316.14 |
| Model fit statistics |  |  |  |  |  |  |  |  |  |
| -2 Log likelihood | 2270.35 | 2270.36 | 2270.44 | 2270.50 | 2270.70 | 2271.05 | 2272.03 | 2273.29 | 2277.25 |
| Cox and Snell R square | 0.02 | 0.02 | 0.02 | 0.02 | 0.02 | 0.02 | 0.02 | 0.02 | 0.02 |
| Nagelkerke R square | 0.12 | 0.12 | 0.12 | 0.12 | 0.12 | 0.12 | 0.12 | 0.12 | 0.12 |
| Hosmer and Lemeshow Chi-square, (P-value) | 16.04 (0.04) | 15.54 (0.05) | 14.82 (0.06) | 12.87 (0.12) | 17.65 (0.02) | 14.38 (0.07) | 20.64 (0.01) | 20.09 (0.01) | 7.71 (0.36) |

X = variables removed in each step
