# Supplementary material for: Rhabdomyolysis among hospitalized patients for salicylate intoxication in the United States: Nationwide inpatient sample 2003–2014
Source: PLoS One. 2021 Mar 8;16(3):e0248242. doi: 10.1371/journal.pone.0248242 (PMC7939294; doi:10.1371/journal.pone.0248242)
Supplement: S3 Table — (DOCX) [file pone.0248242.s004.docx]

**S3 Table** The tests to assess model fit for multiple logistic regression

| Table | Outcome variables | Model fit | | | |
| --- | --- | --- | --- | --- | --- |
|  |  | -2 Log likelihood | Cox and Snell R square | Nagelkerke R square | Hosmer and Lemeshow Chi-square, (P-value) |
| 2 (Backward stepwise method) | Rhabdomyolysis | 2277.25 | 0.02 | 0.12 | 7.71 (0.36) |
| 3 (Enter method) | Invasive mechanical ventilation | 4879.11 | 0.07 | 0.20 | 9.23 (0.32) |
|  | Blood component transfusion | 2154.02 | 0.05 | 0.25 | 7.58 (0.48) |
|  | Renal replacement therapy | 5499.47 | 0.05 | 0.13 | 13.39 (0.10) |
|  | Renal failure | 6738.92 | 0.12 | 0.26 | 14.43 (0.07) |
|  | Respiratory failure | 5743.43 | 0.08 | 0.20 | 14.55 (0.07) |
|  | Circulatory failure | 3614.83 | 0.04 | 0.16 | 10.75 (0.22) |
|  | Liver failure | 1148.28 | 0.01 | 0.12 | 4.15 (0.84) |
|  | Neurological failure | 5171.10 | 0.02 | 0.07 | 12.75 (0.12) |
|  | Hematological failure | 2581.73 | 0.02 | 0.12 | 20.15 (0.01) |
|  | In-hospital mortality | 1276.75 | 0.02 | 0.15 | 6.28 (0.62) |
