# Supplementary material for: Rhabdomyolysis among hospitalized patients for salicylate intoxication in the United States: Nationwide inpatient sample 2003–2014
Source: PLoS One. 2021 Mar 8;16(3):e0248242. doi: 10.1371/journal.pone.0248242 (PMC7939294; doi:10.1371/journal.pone.0248242)
Supplement: S4 Table — (DOCX) [file pone.0248242.s005.docx]

**S4 Table** The tests to assess model fit for multiple linear regression

| Table | Outcome variables | Model fit | | | |
| --- | --- | --- | --- | --- | --- |
|  |  | R | R Square | Adjusted R Square | Standard Error of Estimate |
| 3 (Enter method) | Length of hospital stay (days) | 0.43 | 0.19 | 0.19 | 2.98 |
|  | Hospitalization cost ($) | 0.44 | 0.20 | 0.20 | 26588.09 |
