# Supplementary material for: Rhabdomyolysis among hospitalized patients for salicylate intoxication in the United States: Nationwide inpatient sample 2003–2014
Source: PLoS One. 2021 Mar 8;16(3):e0248242. doi: 10.1371/journal.pone.0248242 (PMC7939294; doi:10.1371/journal.pone.0248242)
Supplement: S5 Table — (DOCX) [file pone.0248242.s006.docx]

**S5 Table** The collinearity test

| Variables in Table 2 | Tolerance | Variance Inflation Factor |
| --- | --- | --- |
| Age | 0.93 | 1.07 |
| Sex | 0.99 | 1.01 |
| Congestive heart failure | 0.95 | 1.05 |
| Atrial flutter/fibrillation | 0.96 | 1.04 |
| Volume depletion | 0.99 | 1.01 |
| Hypokalemia | 0.99 | 1.01 |
| Sepsis | 0.98 | 1.02 |
| Seizure | 0.99 | 1.01 |
| Variables in Table 3 | Tolerance | Variance Inflation Factor |
| Age | 0.72 | 1.39 |
| Sex | 0.97 | 1.03 |
| Race | 0.95 | 1.05 |
| The NIS year | 0.95 | 1.05 |
| Alcohol drinking | 0.96 | 1.04 |
| Anemia | 0.95 | 1.05 |
| Hypertension | 0.73 | 1.37 |
| Dyslipidemia | 0.85 | 1.18 |
| Coronary artery disease | 0.86 | 1.17 |
| Congestive heart failure | 0.92 | 1.08 |
| Atrial flutter/fibrillation | 0.95 | 1.06 |
| Chronic kidney disease | 0.93 | 1.08 |
| Volume depletion | 0.98 | 1.02 |
| Hypokalemia | 0.97 | 1.03 |
| Sepsis | 0.98 | 1.03 |
| Seizure | 0.99 | 1.01 |
| Rhabdomyolysis | 0.98 | 1.03 |
